# Comprehensive Analysis of Expression Regulation for RNA m6A Regulators With Clinical Significance in Human Cancers

**DOI:** 10.3389/fonc.2021.624395

**Published:** 2021-02-23

**Authors:** Xiaonan Liu, Pei Wang, Xufei Teng, Zhang Zhang, Shuhui Song

**Affiliations:** ^1^ National Genomics Data Center, Beijing Institute of Genomics (China National Center for Bioinformation), Chinese Academy of Sciences, Beijing, China; ^2^ School of Future Technology, University of Chinese Academy of Sciences, Beijing, China; ^3^ College of Life Sciences, University of Chinese Academy of Sciences, Beijing, China; ^4^ CAS Key Laboratory of Genome Sciences and Information, Beijing Institute of Genomics, Chinese Academy of Sciences, Beijing, China

**Keywords:** N6-methyladenosine, microRNA, DNA methylation, The Cancer Genome Atlas, prognosis

## Abstract

**Background:**

N6-methyladenosine (m6A), the most abundant chemical modification on eukaryotic messenger RNA (mRNA), is modulated by three class of regulators namely “writers,” “erasers,” and “readers.” Increasing studies have shown that aberrant expression of m6A regulators plays broad roles in tumorigenesis and progression. However, it is largely unknown regarding the expression regulation for RNA m6A regulators in human cancers.

**Results:**

Here we characterized the expression profiles of RNA m6A regulators in 13 cancer types with The Cancer Genome Atlas (TCGA) data. We showed that *METTL14*, *FTO*, and *ALKBH5* were down-regulated in most cancers, whereas *YTHDF1* and *IGF2BP3* were up-regulated in 12 cancer types except for thyroid carcinoma (THCA). Survival analysis further revealed that low expression of several m6A regulators displayed longer overall survival times. Then, we analyzed microRNA (miRNA)-regulated and DNA methylation-regulated expression changes of m6A regulators in pan-cancer. In total, we identified 158 miRNAs and 58 DNA methylation probes (DMPs) involved in expression regulation for RNA m6A regulators. Furthermore, we assessed the survival significance of those regulatory pairs. Among them, 10 miRNAs and 7 DMPs may promote cancer initiation and progression; conversely, 3 miRNA/mRNA pairs in kidney renal clear cell carcinoma (KIRC) may exert tumor-suppressor function. These findings are indicative of their potential prognostic values. Finally, we validated two of those miRNA/mRNA pairs (hsa-miR-1307-3p/*METTL14* and hsa-miR-204-5p/*IGF2BP3*) that could serve a critical role for potential clinical application in KIRC patients.

**Conclusions:**

Our findings highlighted the importance of upstream regulation (miRNA and DNA methylation) governing m6A regulators’ expression in pan-cancer. As a result, we identified several informative regulatory pairs for prognostic stratification. Thus, our study provides new insights into molecular mechanisms of m6A modification in human cancers.

## Introduction

N6-methyladenosine (m6A) is the most abundant modification on eukaryotic mRNA. It plays crucial roles in various biological processes, including neuronal development, spermatogenesis, immune response, cell fate transition, and tumorigenesis (1–5). Dynamic m6A modification is regulated by RNA m6A regulators including methyltransferases, demethylases, and binding proteins, also known as “writers,” “erasers,” and “readers.” METTL3, METTL14, and WTAP are core components of m6A methyltransferase complex (6–8). In addition to the core components, other associated regulatory subunits were also reported in succession, including KIAA1429, ZFP217, RBM15, RBM15B, and CBLL1 (9–11). The m6A demethylases FTO and ALKBH5 can remove m6A mark in the nucleus (2, 12). Several m6A binding proteins have been identified, such as YTH family proteins (YTHDF1/2/3, YTHDC1/2) (13–15) and IGF2BP family proteins (IGF2BP1/2/3) (16–18). Moreover, HNRNPC, HNRNPA2B1, and EIF3A also function as “readers” (19, 20). Overall, it is of great significance to elucidate the potential molecular mechanisms of m6A regulators in distinct biological contexts.

Studies have revealed that m6A modification is of essence in tumorigenesis and progression (e.g., bladder cancer, gliomas, ovarian carcinoma, colorectal carcinoma, hepatocellular carcinoma, clear cell renal cell carcinoma, endometrial cancer, breast cancer, and non-small cell lung cancer) (21–29) by controlling distinct oncogenic pathways. In addition, it has been discovered that m6A regulators have widespread genetic alterations and transcriptional dysregulation in pan-cancer, which can disturb a large number of cancer-related molecular pathways (30). Although the role of m6A modification in oncogenic pathways has been extensively documented in previous studies, the molecular determinants responsible for transcriptional dysregulation of RNA m6A regulators remain unclear. Thus, a deeper understanding is urgently needed.

As known, gene expression is regulated at multiple levels, such as epigenetics, transcription, post-transcription, and post-translation. Among them, microRNA (miRNA) and DNA methylation were widely studied for gene expression regulation (31, 32). Accumulating evidences imply that miRNA can affect the expression of oncogenes and tumor suppressor genes (33–35). For example, hsa-miR-140-5p influences cervical cancer growth and metastasis by targeting *IGF2BP1* (36). In addition, aberrant DNA methylation patterns can also alter gene expression during cancer onset and progression (37–39). For example, hypomethylation of *IGF2BP3* can result in its overexpression in breast cancer (40). Therefore, comprehensive analysis of RNA m6A regulators transcriptional dysregulation from miRNA and DNA methylation levels would be desirable to better understand the underlying mechanisms of m6A expression regulation.

In this study, we first profiled the expression variation map of RNA m6A regulators in multiple cancers. Then, we explored the regulatory roles of miRNA and DNA methylation in m6A regulators transcriptional changes. Moreover, we uncovered several key miRNAs and DNA methylation probes (DMPs). They could not only alter the expression of their corresponding m6A regulators but also act as prognostic predictors. Further analysis of these identified miRNA/mRNA regulatory pairs in kidney renal clear cell carcinoma (KIRC) clearly depicted their associations with cancer progression. Overall, our integrative analysis revealed the upstream regulatory landscape of m6A regulators, which may provide new insights into molecular mechanisms of m6A modification in human cancers and help researchers develop novel targets for cancer diagnosis and treatment.

## Materials and Methods

A bioinformatics pipeline was developed to identify upstream regulatory factors of m6A regulators (**Figure S1**). The detailed methods and tools were described as follows.

### Data Collection and Processing

Multidimensional omics data (including mRNA expression, miRNA expression, and DNA methylation) of The Cancer Genome Atlas (TCGA) cancers and the corresponding clinical data were downloaded from the Broad GDAC Firehose (Stddata_2016_01_28 version, http://gdac.broadinstitute.org/). The mRNA expression data at level 3 in RNA-Seq by expectation maximization (RSEM) format, miRNA expression data in normalized reads per million (RPM) format, 450K DNA methylation array data in *β*-value format, as well as clinical data at level 4 were used for further analysis. To increase the credibility of comparison between tumor and normal samples, primary solid cancers with more than 25 normal samples were retained. The details of all collected datasets used in this study were summarized in **Table S1**.

### Integrative Analysis of miRNA and mRNA Expression Profiles

For miRNA-regulated m6A regulators analysis, the regulatory pairs were downloaded from TargetScan (v7.0, http://www.targetscan.org/) (41) and miRTarBase (v8.0, http://mirtarbase.mbc.nctu.edu.tw/) (42). Thus, for each miRNA/mRNA pair, Spearman correlation analysis was performed using normalized expression values of mRNA-seq and miRNA-seq data. Anti-correlated miRNA/mRNA regulatory pairs (Spearman correlation coefficient (r) < 0, *p*-value < 0.05) were identified in tumor and normal samples, respectively (43, 44). Furthermore, the Wilcoxon rank sum test was used to identify differentially expressed miRNAs and genes (adjusted *p*-value < 0.05), separately. The *p-*value was adjusted by the false discovery rate (FDR) method. The definition of up-regulation (or down-regulation) was that the average expression value of tumor samples was greater (or lower) than that of normal samples. All regulatory pairs, consisting of an up-regulated (or down-regulated) miRNA and its target, a down-regulated (or up-regulated) gene, were screened to build a network with the igraph package in R. The network allowed identifying hub nodes. The nodes with connections greater than or equal to 4 in each cancer were defined as hub genes. The definition of hub miRNA was that the connection of the node was not less than 2 in one cancer. Specifically, the disease and pathway enrichment analyses were performed with the online tool miEAA (v2.0, http://www.ccb.uni-saarland.de/mieaa_tool/) (45). The miRNAs from the network were picked to run miEAA using the miRNA enrichment analysis, in which two categories (disease items from the MNDR database and pathway items from the miRWalk database) were selected with default parameters’ setting. The ggplot2 package in R was used for visualization.

### Integrative Analysis of DNA Methylation and Gene Expression Profiles

To determine the regulation of DNA methylation on m6A regulators, DMPs in the promoter regions (TSS200 and TSS1500) of m6A regulators were selected. Spearman correlation analysis was performed on m6A regulators and their corresponding DMPs (46). As those DMPs are negatively regulating their target genes, anti-correlated regulator pairs (r < 0, *p*-value < 0.05) in tumor and normal samples were obtained. Afterward, differential methylation analysis was performed on DMPs using the ChAMP package in R. The DMPs were defined as hypermethylation (or hypomethylation) when the average *β* value of tumor samples was greater (or lower) than that of normal samples. Only those DMPs satisfying the criteria of FDR < 0.05 were considered as statistically significant (47). All these regulatory pairs were used to construct a biological network. The igraph package in R was used to visualize the regulatory network.

### Identification of Potential Prognostic Regulatory Pairs From the Network

To assess the regulatory pairs with survival outcomes, patients were divided into two groups according to the median value of gene expression or methylation. Patients were defined as high expression or hypermethylation group if their expression or methylation values were greater than the median value. Otherwise, patients were defined as low expression or hypomethylation group. Patient survival between the two groups was assessed via Cox regression analysis. The significance of survival differences was estimated in terms of *p*-value. The regulatory pairs will be considered to have an impact on the prognosis of patients if both *p*-values were lower than 0.05. Kaplan-Meier survival curves were plotted using two R packages (survminer and survival).

### Construction of Prognostic Risk Prediction Model

To acquire the main factors with better prediction effect, the least absolute shrinkage and selection operator (LASSO) Cox regression algorithm was implemented on four potential prognostic regulatory pairs in KIRC with paired miRNA-seq and mRNA-seq data from TCGA. The patients were randomly divided into training dataset (*n* = 200) and test dataset (*n* = 49). The survival and glmnet packages in R were utilized to determine key factors. The risk model was constructed by the following formula:

$$\text{RiskScore}=\sum_{1}^{n}r_{i}\;Exp\;(i)$$

where *r_i_* is regression coefficient, and *Exp*(*i*) is the expression value of the corresponding factor. According to the median value of risk scores, patients were divided into high-risk and low-risk groups respectively. The LASSO regression factor was selected by the minimum value of partial likelihood binomial deviance.

### GO and KEGG Enrichment Analysis

Differentially expressed genes (DEGs) between the high-risk and low-risk groups were determined utilizing the Wilcoxon rank sum test. The functional enrichment analysis of DEGs was performed using DAVID (48). Those terms with *p*-value lower than 0.05 were selected for subsequent analysis. The ggplot2 package was used to visualize the enrichment analysis results. The similarity of these enriched terms was measured with the R package GOSemSim (49).

### Protein-Protein Interaction (PPI) Network Construction

The PPI network was constructed on STRING (v11.0, https://string-db.org/). The key different modules were selected using MCODE in Cytoscape (v3.7.0).

### Immune Infiltration Analysis

The ESTIMATE algorithm was used to calculate the immune score, stromal score, and tumor purity. The marker genes of each immune cell type were collected from previous studies (50). The ssGSEA method (51) was applied to quantify the infiltration degrees of 28 immune cell types in the tumor microenvironment.

## Results

### Comprehensive Expression Analysis Revealed the Prognostic Values of m6A Regulators in Cancers

**Figure 1:**
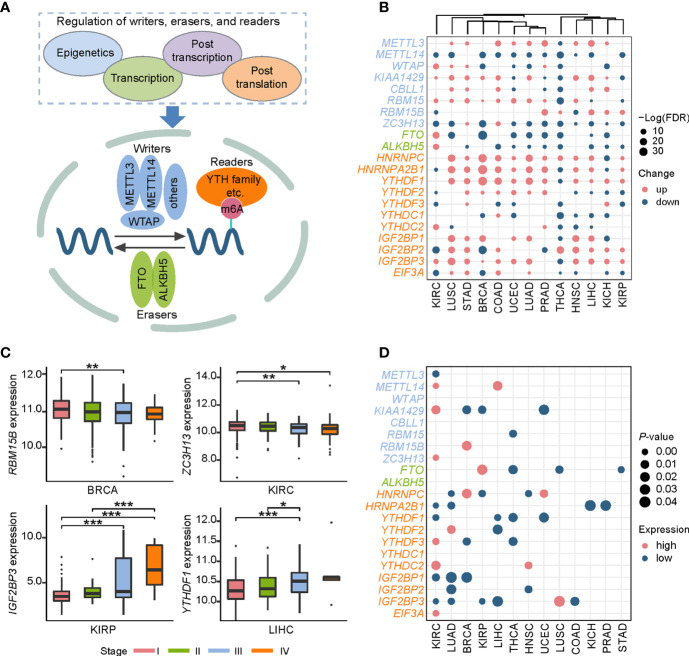
Pan-cancer expression alterations and prognostic values of m6A regulators. **(A)** RNA m6A modification is regulated by RNA m6A regulators, including “writers”-methyltransferase, “erasers”-demethylase, and “readers”-RNA m6A binding proteins. “Writers” consist of core components *METTL3, METTL14, WTAP* and other factors (*KIAA1429, ZFP217, RBM15, RBM15B, and CBLL1*). *FTO* and *ALKBH5* are two “erasers.” “Readers” include *HNRNPC, HNRNPA2B1, YTHDF1, YTHDF2, YTHDF3, YTHDC1, YTHDC2, IGF2BP1, IGF2BP2, IGF2BP3*, and *EIF3A.* **(B)** Expression profiles of RNA m6A regulators in 13 cancer types. Up represents higher expression and down represents lower expression. The circle size represents the statistical significance after controlling FDR. **(C)** Representative examples of expression patterns of m6A regulators across four cancer stages. *P < 0.05, **P < 0.01, and ***P < 0.001. **(D)** Overview of prognostic effects of m6A regulators. High represents the patients with better prognosis when gene expression level is high, and low represents the patients with better prognosis when gene expression level is low.

The dynamic m6A modification is regulated by m6A “writers,” “erasers,” and “readers” (**Figure 1A**). We totally obtained 21 RNA m6A regulators including 8 “writers,” 2 “erasers,” and 11 “readers” through literature curation. We first elucidated the expression characteristics of these regulators in a pan-cancer context (**Figure 1B**): (i) Expression changes of some clusters (*YTHDF* family, *IGF2BP* family, *METTL14*, *FTO*, and *ALKBH5*) were consistent in selected cancers. For example, *YTHDF1* and *IGF2BP3* were up-regulated in 11 cancer types except for THCA. *METTL14* was down-regulated in all 11 cancer types while *FTO* and *ALKBH5* were down-regulated in most cancer types except for KIRC. (ii) Expression alterations of m6A regulators in THCA exhibited a specific pattern among all 13 cancers. Most RNA m6A regulators were significantly down-regulated in THCA except for *RBM15B*, *HNRNPC*, and *IGF2BP2*. These findings suggest that there are multiple mechanisms capable of controlling gene expression of m6A regulators in distinct cancers.

Combined with clinical data, we further investigated expression patterns of all m6A regulators in four different cancer stages (stage I, stage II, stage III, and stage IV), a widely used signature for predicting the outcomes of patients (**Table S2**). Two patterns significantly associated with cancer staging were observed: a decreased expression level of *RBM15B* in breast invasive carcinoma (BRCA) and *ZC3H13* in KIRC was accompanied by the progression of cancer stages, while *YTHDF1* in liver hepatocellular carcinoma (LIHC) and *IGF2BP3* in kidney renal papillary cell carcinoma (KIRP) showed the increased expression pattern (**Figure 1C**). Since cancer staging is primarily defined by clinicopathologic features, these observations suggest that m6A regulators may influence patients’ survival. Furthermore, we depicted a landscape for strongly survival-related genes across 13 cancer types, and then identified several potential oncogenes and tumor suppressor genes (**Figure 1D** **)**. For instance, *IGF2BP1* and *IGF2BP3* showed an oncogenic role in KIRC and lung adenocarcinoma (LUAD). While *METTL14* and *YTHDC2* functioned as tumor suppressors in KIRC. Both the IGF2BP family proteins, METTL14, and YTHDC2 can function in cancers through directing m6A-modified mRNAs. Together, these results indicate that m6A regulators can be used to develop novel treatment strategies.

### Identification of miRNAs Targeting m6A Regulators in Pan-Cancer

**Figure 2:**
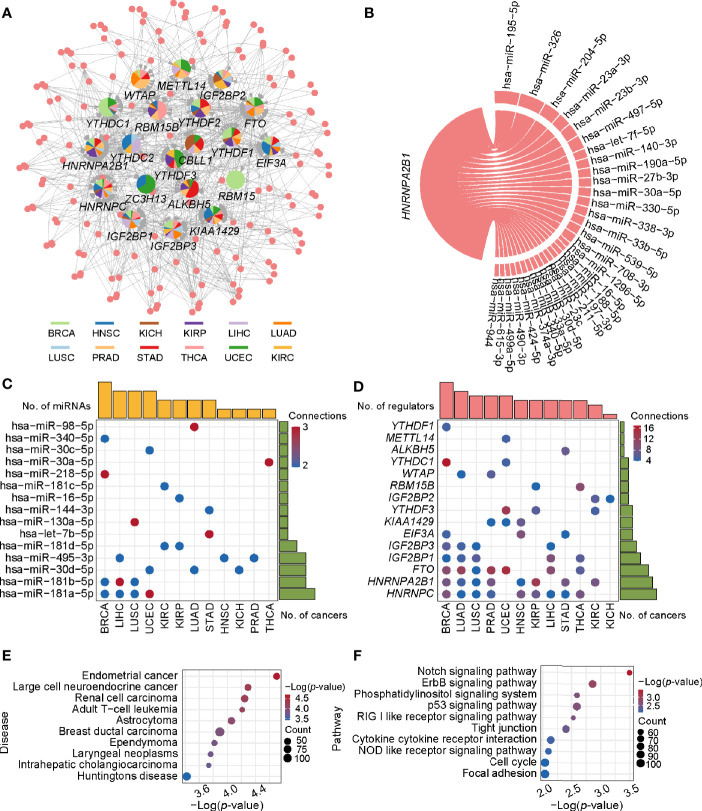
The regulatory network and enriched pathways of miRNA-m6A regulators. **(A)** The regulatory network of miRNAs and m6A regulators in pan-cancer. In the pie chart, different colors represent different cancers, and size reflects the number of regulatory pairs. The circle represents miRNAs. The m6A regulators’ names were labeled. **(B)** The *HNRNPA2B1* associated regulatory pairs in the pan-cancer network. The line width represents the number of cancers with this regulatory pair. **(C)** Statistics of hub miRNAs in 12 cancer types. When the connection of miRNA node in the network is greater than or equal to 2, the node is defined as hub miRNA. The top bar out of chart represents the number of hub miRNAs for each cancer and the right bar indicates the number of cancers for each miRNA. The redder the color, the more the connections. **(D)** Statistics of hub genes in 12 cancer types. When the connection of gene node is greater than or equal to 4, the node is defined as hub gene. The top bar out of chart is the number of hub regulators for each cancer. The right bar presented the number of cancers for each regulator. **(E)** Disease enrichment analysis of miRNAs. **(F)** Pathway enrichment analysis of miRNAs.

As mentioned above, the expression of m6A regulators had a significant difference between tumor and normal samples. Thus, in what follows, we aimed to investigate their upstream regulatory factors that can regulate the expression of these genes. From 1,255 predicted and experimentally confirmed miRNA/mRNA regulatory pairs, 629 regulatory pairs showing negative correlation (r < 0) across 12 cancer types were selected for further analysis. Among them, 45% (282 out of 629) significantly differentially expressed (*p*-value < 0.05; tumor vs normal) pairs (consisting of 158 miRNAs and 20 m6A regulators) (**Table S3**) were used to construct a pan-cancer miRNA-gene regulatory network (**Figure 2A**). The network showed some observations: i) *RBM15*-associated regulatory pairs were only identified in BRCA. ii) *HNRNPC*-associated regulatory pairs were presented in 11 cancer types, of which BRCA had the most 9 regulatory pairs. iii) *HNRNPA2B1* had the maximum connection. The hsa-miR-195-5p and hsa-miR-326 regulating *HNRNPA2B1* were found in more than one cancer type (**Figure 2B**). Next, we picked out all the hub miRNAs and genes (see methods for details) involving in the transcriptional regulatory network. A case in point is hsa-miR-181a-5p belonging to miR-181 family can target several m6A regulators in BRCA, LIHC, LUSC, and UCEC (**Figure 2C**). The hsa-miR-181a-5p has been reported to be associated with acute myeloid leukemia, papillary thyroid cancer, endometrial carcinoma and so on (52–54). Some m6A regulators, such as *HNRNPC*, *HNRNPA2B1*, and *FTO*, can also be targeted by several miRNAs (**Figure 2D**). In addition, statistical analysis of the network showed that 159 regulatory pairs were found in only one cancer type and 13 regulatory pairs were found in at least 5 cancer types (**Table S4**). These results indicate that these miRNAs may play important roles in expression alterations of m6A regulators.

To further understand the functional characteristic of miRNAs in the regulatory network, we performed miRNA enrichment analysis. Among disease ontology items, they were significantly associated with several cancers (such as endometrial cancer, renal cell carcinoma, and breast ductal carcinoma) (**Figure 2E**). In addition, results from miEAA revealed that the candidate set of miRNAs was enriched in some pathways associated with cancer, immune and cellular processes, such as p53 signaling pathway, RIG I like receptor signaling pathway, and cell cycle (**Figure 2F**). More importantly, 13 of the above regulatory pairs have been reported in published studies (**Table 1**). For example, hsa-miR-145 could regulate the expression of *YTHDF2* in hepatocellular carcinoma, which further affected the m6A modification and promoted the disease progression (33). Another example, hsa-miR-188 could inhibit the proliferation, migration and invasion of glioma by suppressing the expression of *IGF2BP2* (55).

**Table 1:** Regulatory relationships with literature evidence.

miRNA		Gene	PMID	Journal	Disease	TCGA	m6A
hsa-miR-497	*EIF3A*		28322466	J Cell Biochem.	Pulmonary fibrosis	LIHC	–
hsa-miR-30b-5p	*FTO*		31728912	J Physiol Sci.	Hypoglycemia-associated autonomic failure	KICH, STAD, UCEC	–
hsa-miR-495	*FTO*		31709454	Pflugers Arch.	Type 2 diabetes	KIRC, STAD	–
hsa-miR-30a-5p	*FTO*		31728912	J Physiol Sci.	Hypoglycemia-associated autonomic failure	KIRP	–
hsa-miR-491-5p	*IGF2BP1*		27158341	Am J Transl Res.	Non-small cell lung cancer	LIHC	–
hsa-miR-150	*IGF2BP1*		26561465	Tumour Biol.	Osteosarcoma	KIRP	–
hsa-miR-150	*IGF2BP1*		30220021	Pathol Oncol Res.	Osteosarcoma	KIRP	–
hsa-miR-98-5p	*IGF2BP1*		28244848	Oncol Res.	Hepatocellular carcinoma	LIHC	–
hsa-miR-140-5p	*IGF2BP1*		27588393	Oncotarget.	Cervical cancer	KIRP	–
hsa-let-7b	*IGF2BP2*		27513293	Exp Dermatol.	Wound healing	HNSC, LUSC, STAD	–
hsa-miR-188	*IGF2BP2*		28901413	Mol Med Rep.	Glioma	KIRC	–
hsa-miR-145	*YTHDF2*		28104805	J Biol Chem.	Hepatocellular carcinoma	BRCA, THCA	m6A
hsa-miR-106b-5p	*YTHDF3*		30341748	Breast Cancer.	Breast cancer	KICH, LUSC, UCEC	–

**Figure 3:**
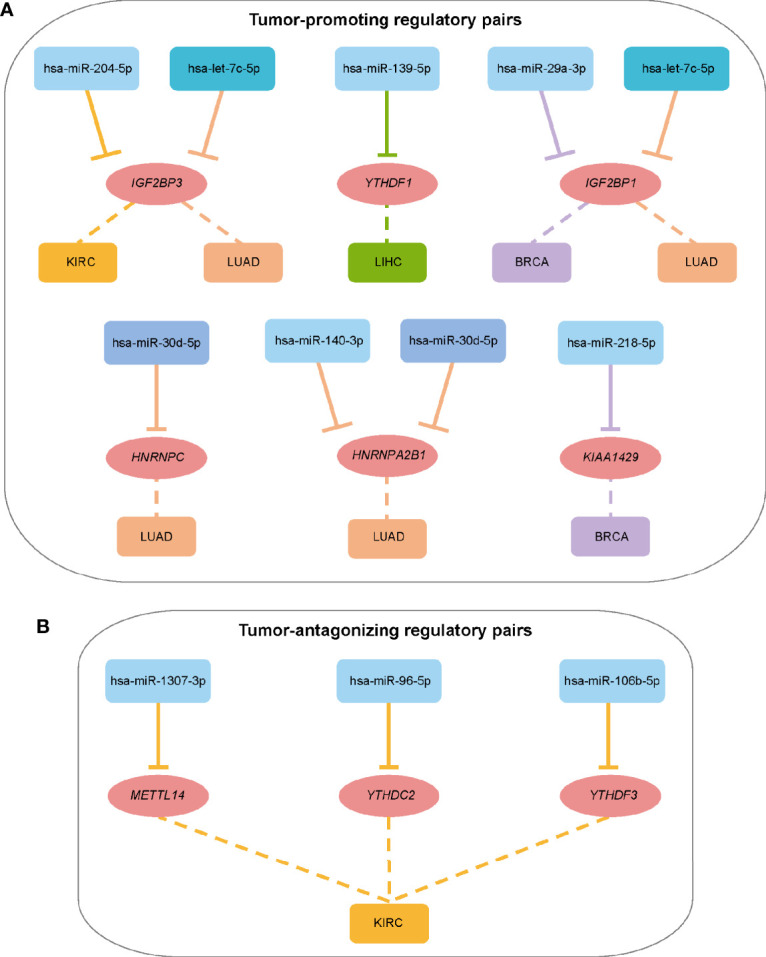
Summary of regulatory relationships between miRNAs and m6A regulators that potentially function as tumor-promoting **(A)** and tumor-antagonizing regulatory pairs **(B)**. Lines of the same color represent the same type of cancer.

Survival analysis identified some miRNA/mRNA regulatory pairs with prognostic value (**Figures S2–S4** **)**. Taken the hsa-miR-204-5p/*IGF2BP3* pair in KIRC for example, low expression of *IGF2BP3* and high expression of hsa-miR-204-5p exhibited a favorable outcome. Therefore, this regulatory pair was defined as a tumor-promoting pair. As for hsa-miR-96-5p/*YTHDC2*, high expression of *YTHDC2* and hsa-miR-96-5p exhibited favorable and opposite outcome respectively, which was thus defined as a tumor-antagonizing pair. Totally, 12 prognosis-related miRNA/mRNA regulatory pairs (9 tumor-promoting and 3 tumor-antagonizing pairs) in four cancer types were finally obtained (**Figure 3**). Besides, several miRNAs including hsa-miR-204-5p, hsa-miR-1307-3p, hsa-miR-96-5p, and hsa-miR-106b-5p may affect the survival and prognosis of patients by regulating the expression of *IGF2BP3*, *METTL14*, *YTHDC2*, and *YTHDF3*, respectively, in KIRC; hsa-let-7c-5p may target multiple m6A regulator genes (including *IGF2BP1* and *IGF2BP3*) in LUAD. Together, those identified miRNAs can account for the differential expression of m6A regulators, and they can serve as potential targets for cancer therapy.

### DNA Methylation Probes (DMPs) Targeting m6A Regulators Are Predictive of Patients’ Outcome

**Figure 4:**
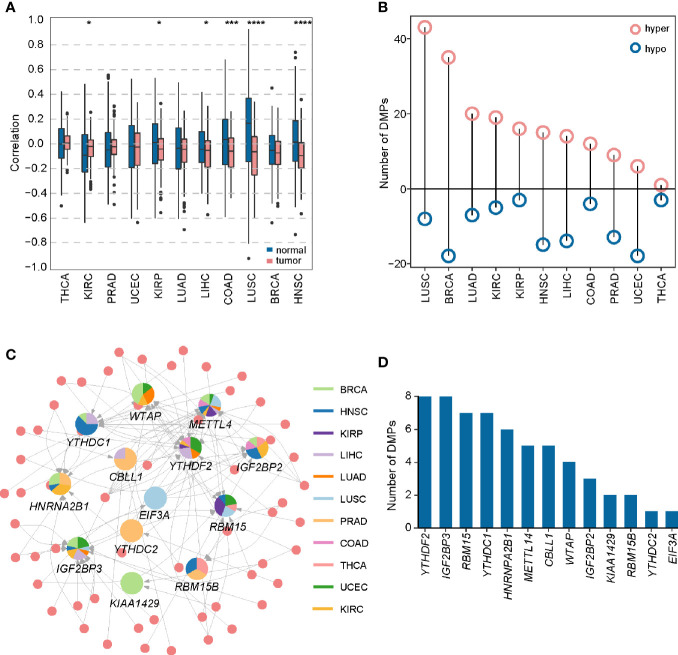
Construction of DMP-mRNA regulatory network. **(A)** Boxplot of Spearman’s correlation between DNA methylation data and mRNA-seq data across 11 cancer types. *P < 0.05, ***P < 0.001, and ****P < 0.0001. **(B)** The number of differentially methylated probes in different cancer types. **(C)** The regulatory network of DNA methylation probes and m6A regulators in pan-cancer. In the pie chart, different colors represent different cancers, and size reflects the number of regulatory pairs. The circle represents DMPs. **(D)** Statistics of the number of DMPs regulating m6A regulators in the pan-cancer regulatory network.

DNA methylation, an extensively studied epigenetic mark, can affect transcriptional dysregulation in cancers (56). Then, we addressed the effect of DNA methylation on m6A regulators transcriptional dysregulation. Spearman correlation analysis showed that DMPs were negatively correlated with their target genes in most cancers **(** **Figure 4A** **)**, except that THCA exhibited minor differences between positive and negative correlations in both tumor and normal tissues. Totally, we identified 154 regulatory pairs showing the negative correlation across 11 cancer types. Among the 154 regulatory pairs, 58 unique DMPs were differential methylation. We detected much more frequent hypermethylation than hypomethylation in most cancers (**Figure 4B**). Collectively, most DMPs were hyper-methylated and negatively regulated their target genes (m6A regulators) in a pan-cancer layer. These results indicate that DNA methylation can also account for m6A expression alterations in cancers.

**Figure 5:**
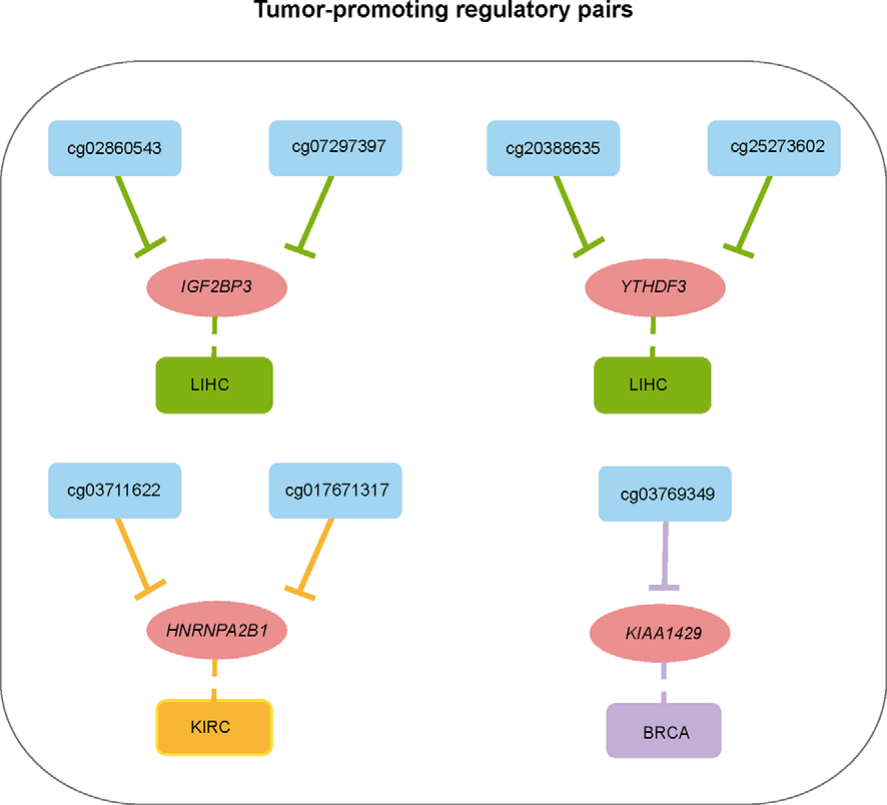
Summary of regulatory relationships between DNA methylation probes and m6A regulators that potentially affect patients prognosis in cancers.

To show a landscape for all potential DMP/gene regulatory pairs across 11 cancer types, we further built a regulatory network (**Figure 4C**) using 100 anti-correlated regulatory pairs, involving 58 differentially methylated DMPs and 13 differentially expressed m6A regulators (**Table S5**). The network showed that *METTL14* was targeted by multiple DMPs in most cancer types. Oppositely, *KIAA1429*, *YTHDC2*, and *EIF3A* associated pairs were only found in one cancer. Based on statistical analysis of the network, we found that 33 regulatory pairs occurred only in one cancer, and 13 regulatory pairs presented in at least three cancer types (**Table S6**). In addition, we also found that *IGF2BP3* and *YTHDF2* were regulated by eight different DMPs across six cancers (**Figure 4D**). Subsequent survival analysis identified seven regulatory relationships, which may serve as tumor-promoting regulatory pairs (**Figure 5**). For example, *IGF2BP3* targeted by cg02860543 and cg07297397 could affect the survival and prognosis of patients in LIHC. Two methylation probes (cg03711622 and cg17671317) could target *HNRNPA2B1* in KIRC. The Kaplan-Meier curves showed that the expression and methylation levels of patients with better outcome were the opposite (**Figure S5**). Our findings indicate that m6A regulators with clinical significance in human cancers can be influenced by dynamic DNA methylation.

### Potential Application of miRNA-m6A Regulator Pairs in KIRC Prognosis

**Figure 6:**
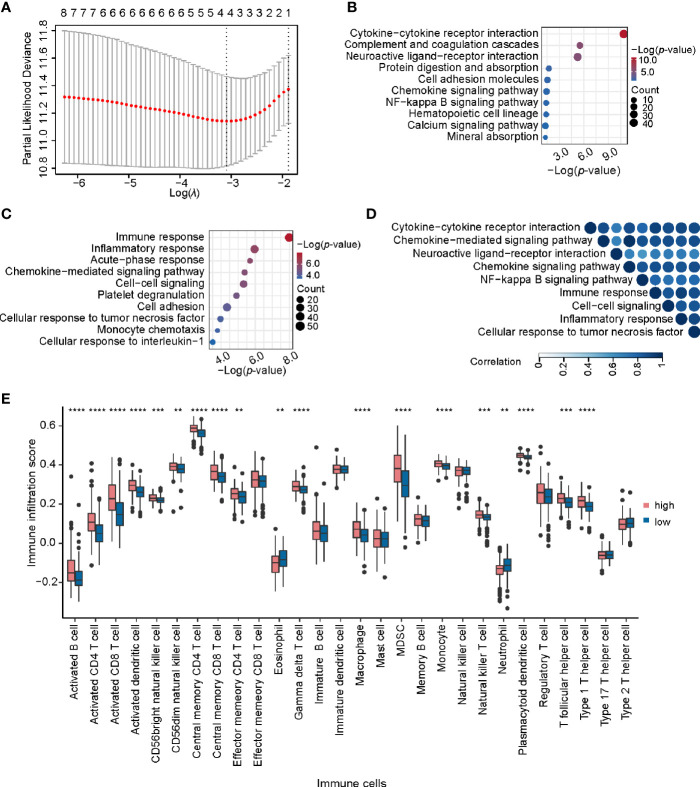
Identification and analysis of key regulatory pairs in KIRC. **(A)** The process of building the signature using LASSO regression algorithm. **(B)** Biological process enrichment analysis of differentially expressed genes between high-risk and low-risk groups. **(C)** KEGG pathway enrichment analysis of differentially expressed genes in high-risk and low-risk groups. **(D)** Functional similarity analysis of gene sets in terms of biological processes. The order of enrichment items in the upper part of triangle is consistent with that of the left side. **(E)** Comparison of infiltration scores of 28 immune cell types between high-risk and low-risk groups. **P < 0.01, ***P < 0.001, and ****P < 0.0001.

To further explore the potential application of miRNA/mRNA regulatory pairs, subsequent in-depth analyses were focused on KIRC. We wonder whether there are any key regulators in specific cancer type. Based on 4 regulatory pairs (hsa-miR-1307-3p/*METTL14*, hsa-miR-106b-5p/*YTHDF3*, hsa-miR-96-5p/*YTHDC2*, and hsa-miR-204-5p/*IGF2BP3*) identified above in KIRC, we screened prognostic regulatory pairs that could best separate risk groups using LASSO regression analysis (**Figure 6A**). The most appropriate number of factors was 4 when the partial likelihood binomial deviance reached the minimum value. Then the four factors (hsa-miR-1307-3p, *METTL14*, hsa-miR-204-5p, and *IGF2BP3*, composed two regulatory pairs hsa-miR-1307-3p/*METTL14* and hsa-miR-204-5p/*IGF2BP3*) were selected to construct the prediction model (see details in *Materials and Methods*). Next, patients’ risk score was imputed by the expression values and regression coefficients of these 4 factors. The risk score was used to divide the patients into high-risk and low-risk groups, of which the low-risk group was associated with better survival (*p*-value < 0.0001). Similar findings were also observed in additional validation dataset (**Figures S6**). These results disclose that expression profiles of hsa-miR-1307-3p/*METTL14* and hsa-miR-204-5p/*IGF2BP3* pairs can well characterize the survival status of patients in KIRC.

We further identified 1,314 DEGs in high-risk group against the low-risk group, including 267 up-regulated and 1047 down-regulated genes. KEGG pathway enrichment analysis of these DEGs detected multiple immune-related pathways (including complement and coagulation cascades, hematopoietic cell lineage, and chemokine signaling pathway) (**Figure 6B**). In addition, pathways related to signal transduction were enriched, such as cytokine-cytokine receptor interaction, neuroactive ligand-receptor interaction, and cell adhesion molecules, etc. Meanwhile, Gene Ontology (GO) enrichment analysis also showed that these DEGs were related to immunity and signal transduction, such as immune response, cell-cell signaling, chemokine-mediated signaling pathway, and inflammatory response (**Figure 6C** **).** The similarity matrix of enriched terms (**Figure 6D**) further confirmed that immune-related terms presented high similarities with those terms related to signal transduction or other processes, such as immune response and cytokine-cytokine receptor interaction. Moreover, we constructed the PPI network for DEGs with clinical significance and identified four important modules (**Figure S7A**). Of note, several immune-related genes were found in those PPI modules, such as chemokine family (*CXCL8*, *CXCL4*, *CXCL6, CCL5*, and *C3*), interleukin (*IL1A* and *IL6*) and so on. These results indicate that these regulatory pairs may function through immune-related mechanisms.

As the functional classes of DEGs were mainly related to immunity, we further calculated the immune score, stromal score, and tumor purity of samples belonging to each risk group. It is worth mentioning that the high-risk group had higher immune score and lower tumor purity by comparison with the low-risk group (**Figure S7B**). Recent studies found that m6A regulators were closely correlated with immune infiltration in glioma and gastric cancer (57, 58), and thus we wondered whether the immune infiltration was different between the two groups. Most immune cells have significantly higher infiltration score in the high-risk group than low-risk group (**Figure 6E**). From the correlation analysis between m6A regulators’ expression and immune cell infiltration score (**Table S7**), we found that the expression of *IGF2BP3* was positively correlated with the infiltration scores across 11 immune cells, suggesting that highly expressed *IGF2BP3* may contribute strong immune infiltration and poor survival. In short, we speculate that hsa-miR-204-5p may affect the immune-related processes and immune infiltration by regulating *IGF2BP3.* Such a regulatory axis may promote the occurrence and development of KIRC.

## Discussion

With more effective sequencing technologies and tools (59–61), how dysregulated m6A is involved in cancer pathogenesis and progression has attracted much more attention than ever. Here, we profiled the expression variation map of RNA m6A regulators in multiple cancers and explored the upstream regulation of m6A regulators from miRNA and DNA methylation. Furthermore, we identified the potential miRNA-regulated and DNA methylation-regulated regulatory pairs and investigated the effects of miRNA/mRNA regulatory pairs on patients in KIRC.

Till now, a few of studies have showed that transcriptional dysregulation of m6A regulators in pan-cancer (30). Here we reported the altered expression of RNA m6A regulators across 13 cancer types in comparison with normal samples, revealing two rules in expression dynamics: the expression of “reader” proteins IGF2BP family and YTHDF family were up-regulated in most cancers, while methyltransferase METTL14, demethylase FTO and ALKBH5 were down-regulated in most cancers. Besides, these varied expression levels were correlated with survival advantages or disadvantages. Although some of them have been reported to play an oncogenic or tumor-suppressive role in different cancers, the role of m6A regulators was only involved in the regulation of cancer-related gene expression (62). The reasons of m6A regulators dysregulation were unclear. As we know, miRNA (63) and DNA methylation (64) are two essential modulation for controlling gene expression, and a large amount of miRNA and DNA methylation sequencing data have been generated. Correlation analysis was first performed on individual m6A regulators for methylation and expression. Then, differential expression and methylation analysis were performed on individual miRNA and DMP. We built regulatory networks with identified potential miRNA/gene and DMP/gene regulatory pairs, in which some pairs had been reported to exert positive effect on cancer pathogenesis and progression. For example, hsa-miR-150/*IGF2BP1* regulatory pair was reported to be a novel potential therapeutic target for osteosarcoma treatment (65), and *IGF2BP1* was identified as a novel target gene of hsa-miR-98-5p in hepatocellular carcinoma (66). Similarly, the DNA demethylation in the promoter region of *IGF2BP3* could influence the progression of G-CIMP gliomas (67), and cg07166550/*ALKBH5* could be used as prognostic biomarkers in prostate cancer (68). Finally, we identified some regulatory pairs with prognostic significance in several cancers. Moreover, studies have reported that differential expression or methylation is highly related with tumorigenesis through regulating gene expression (69–72). Our study identified miRNAs/probes that were differentially expressed/methylated between tumor and normal samples, indicating their potential association with tumorigenesis. Based on four cancer stages, we found that two miRNAs and one probe were relevant to tumor progression (**Figure S8**). Among them, a decreased expression of hsa-miR-204-5p in KIRC and cg03769349 in LIHC was accompanied by the progression of cancer stages, while hsa-miR-106b-5p in KIRC showed the opposite pattern. These findings suggest that miRNA or DNA methylation can affect the tumorigenesis and progression. In addition, when searching for BBcancer (http://bbcancer.renlab.org/; 73), we found that each member of *YTHDC2/*hsa-miR-96-5p regulatory pair had higher expression abundance in peripheral blood. This finding suggests that this regulatory pair can serve as a biomarker for early diagnosis of cancers. All these studies indicate that the detailed mechanisms of miRNA-mRNA and DMP-mRNA regulatory pairs in human cancers warrant further investigation.

Renal cell carcinoma (RCC) is the most lethal urogenital tumor, among which clear cell RCC (ccRCC, also known as KIRC) constitutes 70% to 80% of all RCCs. Few studies found that the prognostic value of some m6A regulators in KIRC (74), but the detailed mechanisms remained unclear. Here we totally identified four miRNA/mRNA regulatory pairs (hsa-miR-1307-3p/*METTL14*, hsa-miR-106b-5p/*YTHDF3*, hsa-miR-96-5p/*YTHDC2*, and hsa-miR-204-5p/*IGF2BP3*) in KIRC. For the four regulatory pairs, we verified the expression relationship of these regulatory pairs using an independent dataset from GEO. As a result, we did find hsa-miR-106b-5p/*YTHDF3* regulatory pair in GSE16441. This finding makes our analysis more credible. To explore the potential application of them in KIRC prognosis. We first performed LASSO Cox regression analysis and identified two regulatory pairs (including hsa-miR-1307-3p/*METTL14* and hsa-miR-204-5p/*IGF2BP3*) in KIRC as significant prognosis-related pairs. The role of *METTL14* and *IGF2BP3* in human cancers was studied before. The promotion function by *METTL14* in pancreatic cancer was uncovered (75) and *IGF2BP3* was found to be a potential prognosis marker and therapeutic target of colon cancer (76). Yet the miRNA-mediated mechanisms of *METTL14* and *IGF2BP3*, if any, remain unclear. According to the expression level of these two pairs, we built a risk model to divide the patients into high-risk and low-risk groups. We found that DEGs between high-risk and low-risk groups were enriched in immune-related biological processes. Moreover, the infiltration score of 28 kinds of immune cells in tumor tissues showed statistically different patterns in the two risk groups. Notably, the expression level of *IGF2BP3* had a strong positive correlation with the infiltration scores of multiple immune cells, suggesting that different features of tumor infiltration may contribute by the expression change of *IGF2BP3*.

In summary, our study demonstrated that miRNA- or DNA methylation- regulated m6A regulators expression involved in tumor progression and strongly correlated with patients’ prognosis. Although three types of sequencing data (miRNA-seq, mRNA-seq, and methylation array data) from TCGA were used in our study, a large-scale and multi-omics (such as CNV, lncRNA, and proteomic data) integrative analysis would be desirable as future directions. Furthermore, validation experiments are highly needed to convince our results in the future. Accordingly, all these data should be integrated to build a multi-dimensional regulatory network for better understanding the complex mechanisms of m6A regulators in cancers.

## Data Availability Statement

The original contributions presented in the study are included in the article/**Supplementary Material**. Further inquiries can be directed to the corresponding authors.

## Author Contributions

SS: conception and design. SS, ZZ, and XT: writing—review and editing. XL, PW, and XT: methodology. XL and PW: formal analysis. XL and XT: writing—original draft. All authors contributed to the article and approved the submitted version.

## Funding

SS received funding support from The Youth Innovation Promotion Association of Chinese Academy of Science (2017141). The Strategic Priority Research Program of the Chinese Academy of Sciences (Grant No. XDA19090116 to SS, Grant No. XDA19050302 to ZZ).

## Conflict of Interest

The authors declare that the research was conducted in the absence of any commercial or financial relationships that could be construed as a potential conflict of interest.

## Abbreviations

BRCA, breast invasive carcinoma; COAD, colon adenocarcinoma; HNSC, head and neck squamous cell carcinoma; KICH, kidney chromophobe; KIRC, kidney renal clear cell carcinoma; KIRP, kidney renal papillary cell carcinoma; LIHC, liver hepatocellular carcinoma; LUAD, lung adenocarcinoma; LUSC, lung squamous cell carcinoma; PRAD, prostate adenocarcinoma; STAD, stomach adenocarcinoma; THCA, thyroid carcinoma; UCEC, uterine corpus endometrial carcinoma; m6A, N6-methyladenosine; TCGA, The Cancer Genome Atlas; DMPs, DNA methylation probes; ssGSEA, single sample gene set enrichment analysis; KEGG, Kyoto Encyclopedia of Genes and Genomes.
