# Supplementary figures and images for: Comprehensive Analysis of Expression Regulation for RNA m6A Regulators With Clinical Significance in Human Cancers
Source: Front Oncol. 2021 Feb 23;11:624395. doi: 10.3389/fonc.2021.624395 (PMC7946859; doi:10.3389/fonc.2021.624395)

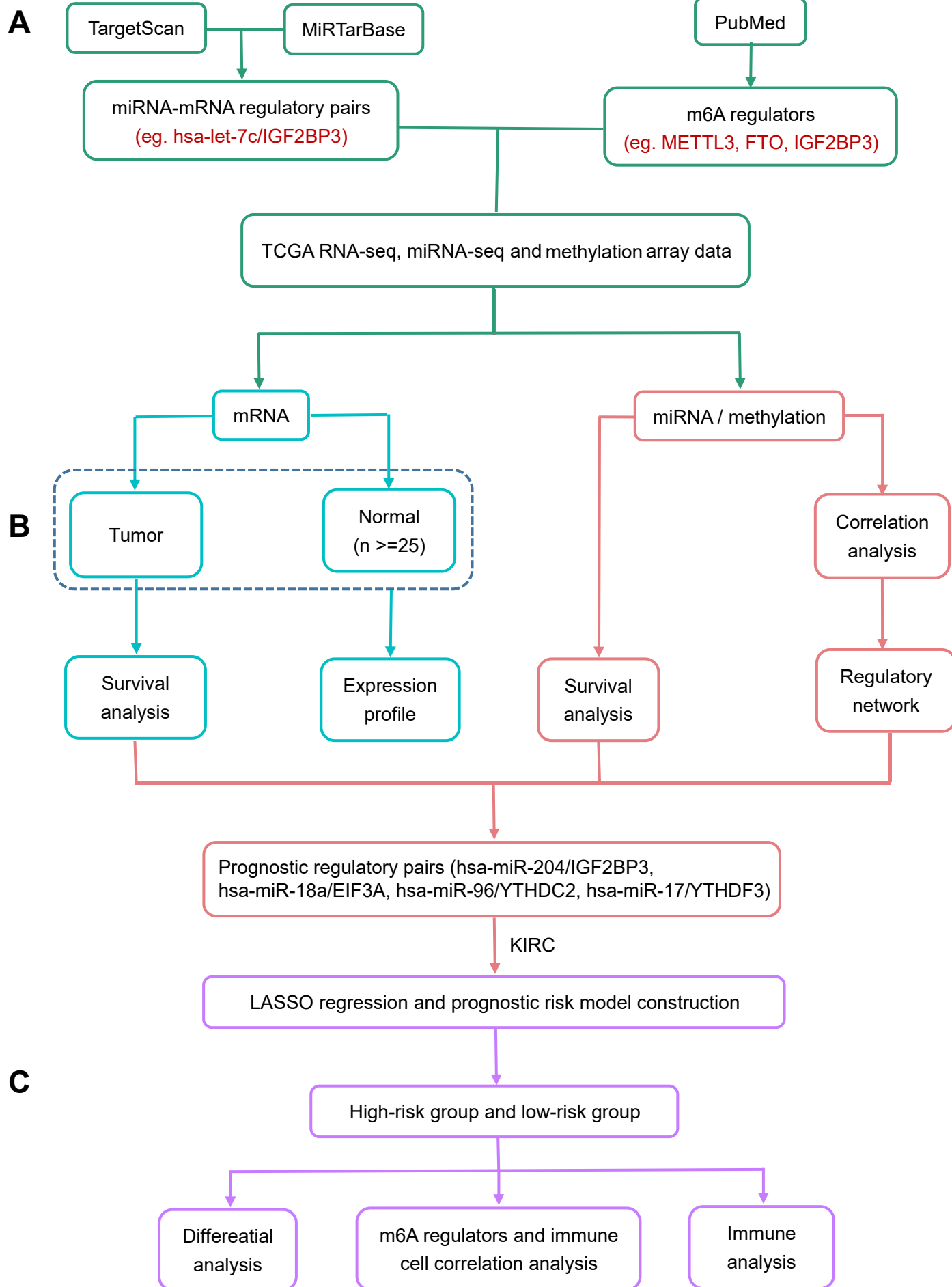

Supplement: Supplementary file 1 [file DataSheet_1.zip › Supplementary Figures/Figure S1.pdf]

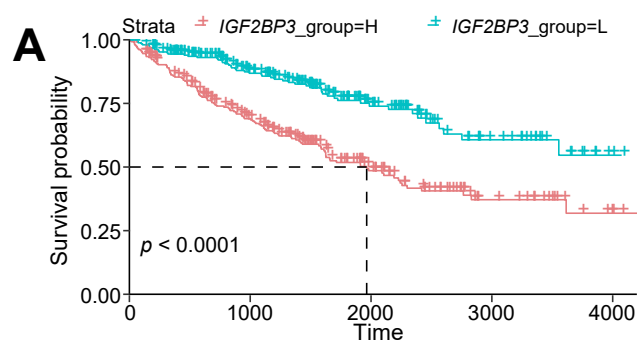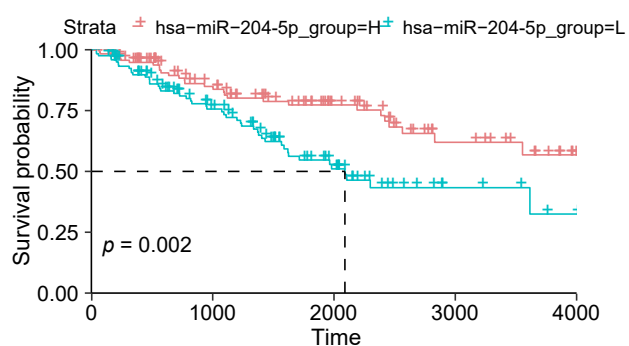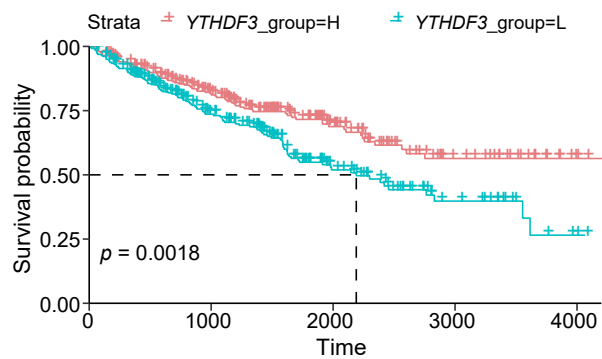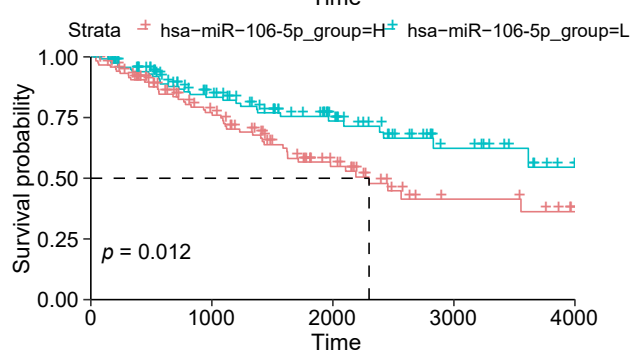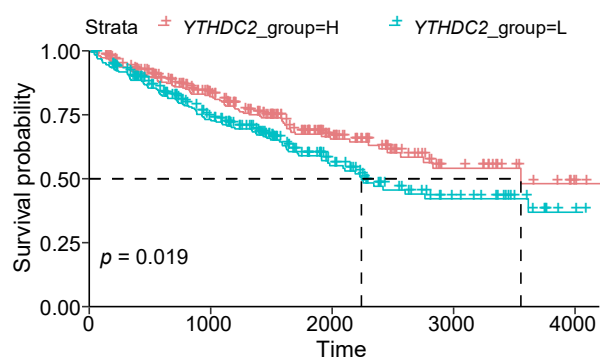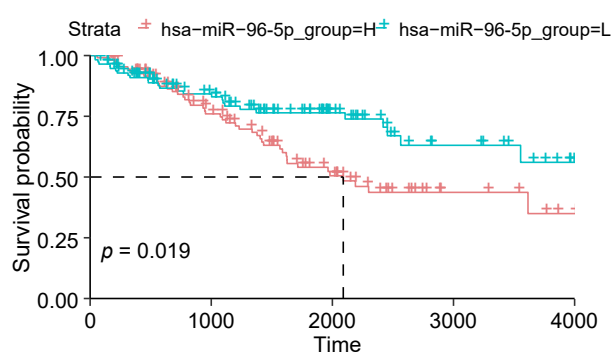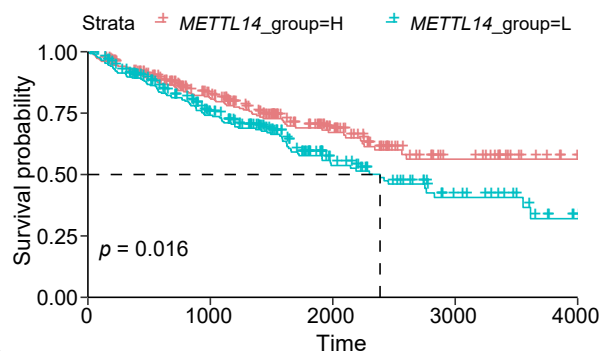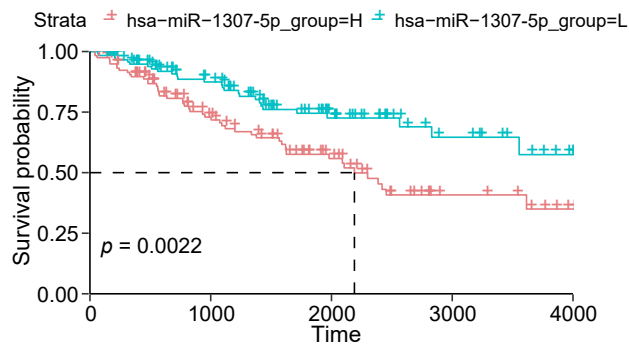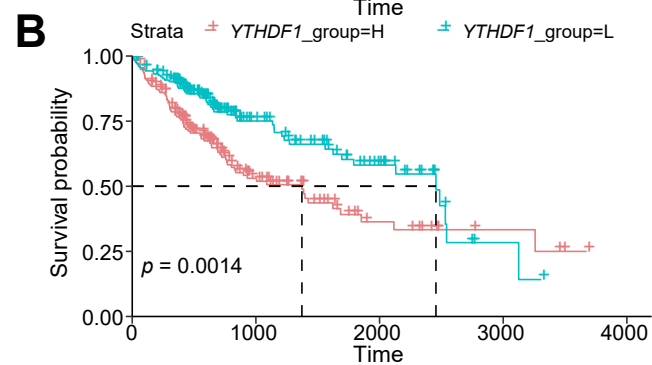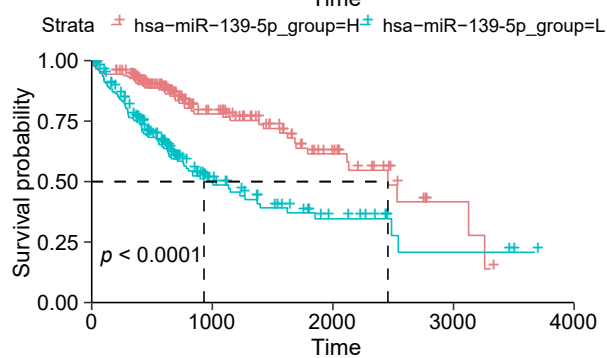

Supplement: Supplementary file 1 [file DataSheet_1.zip › Supplementary Figures/Figure S2.pdf]

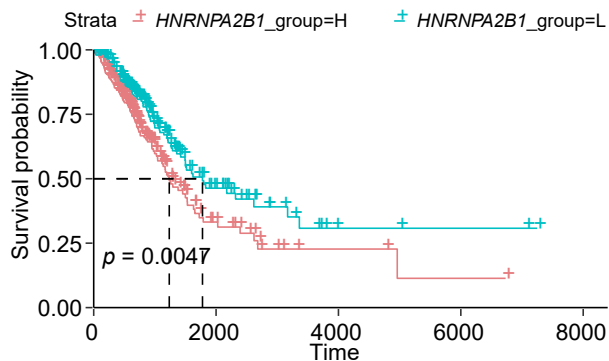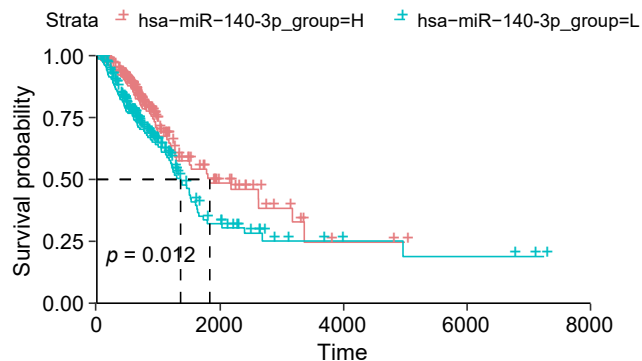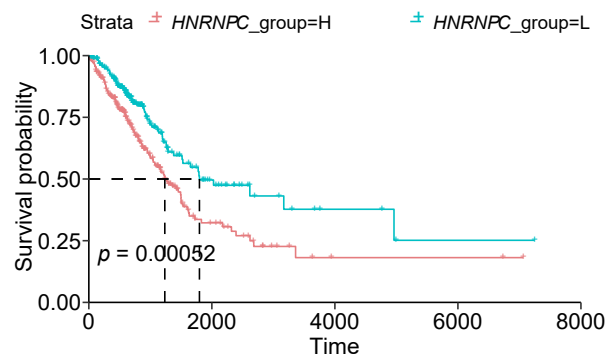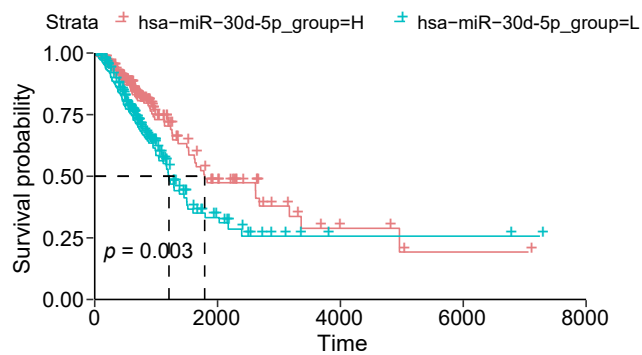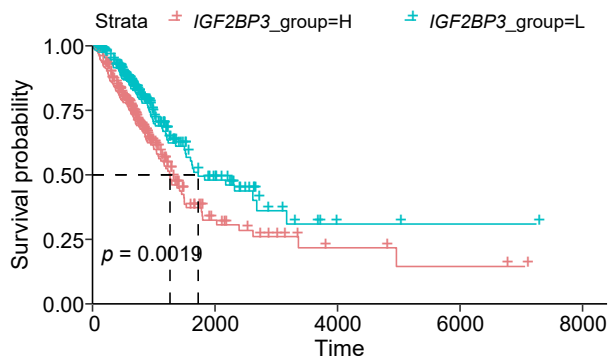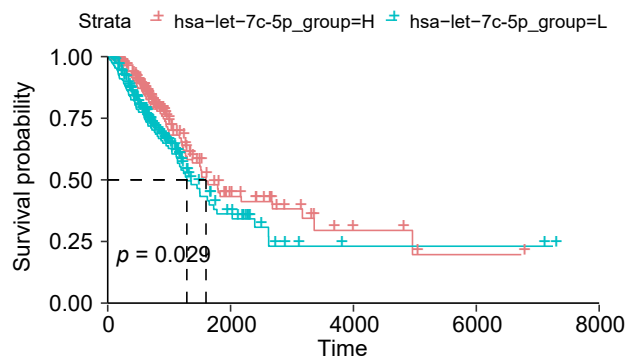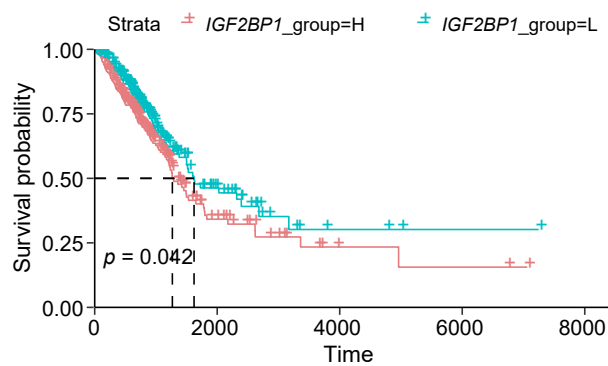

Supplement: Supplementary file 1 [file DataSheet_1.zip › Supplementary Figures/Figure S3.pdf]

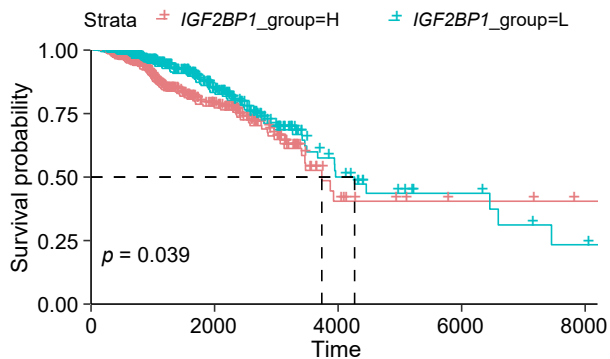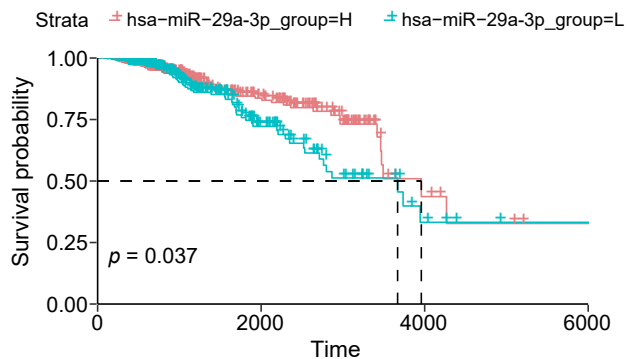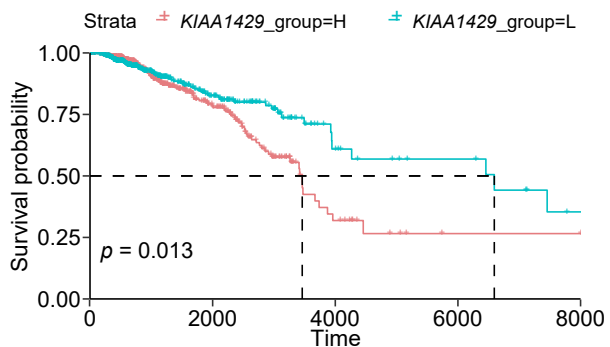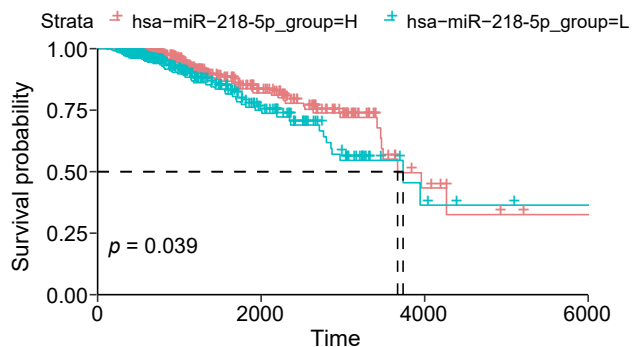

Supplement: Supplementary file 1 [file DataSheet_1.zip › Supplementary Figures/Figure S4.pdf]

**A**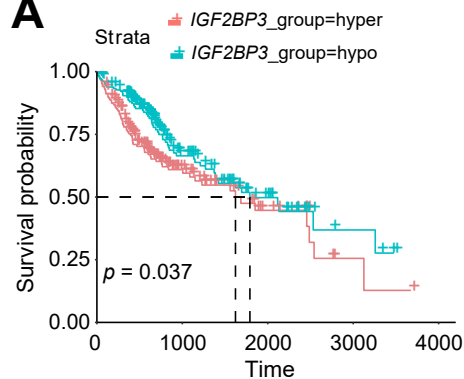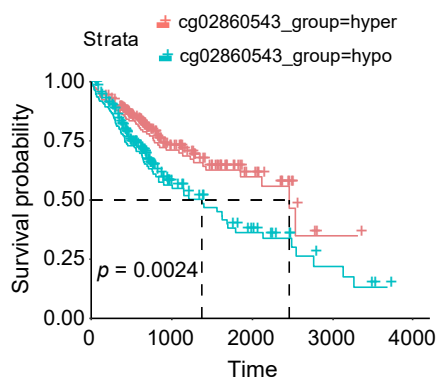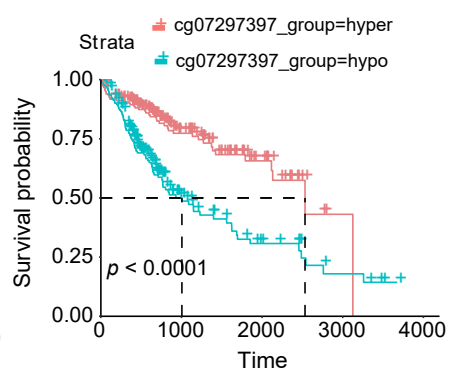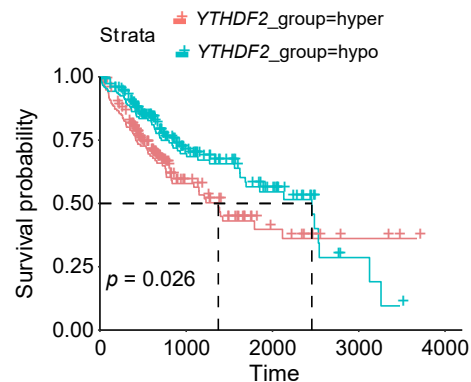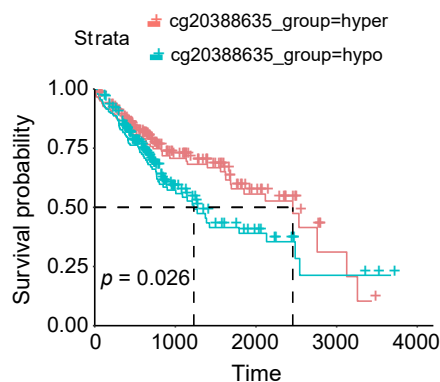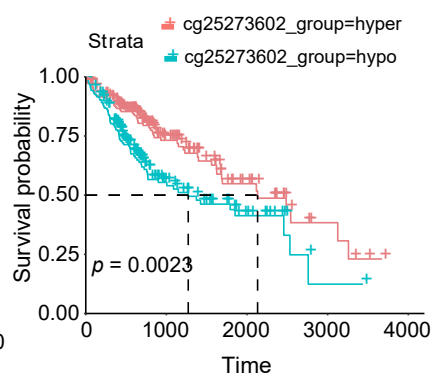**B**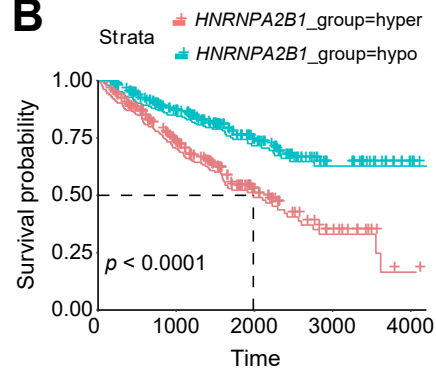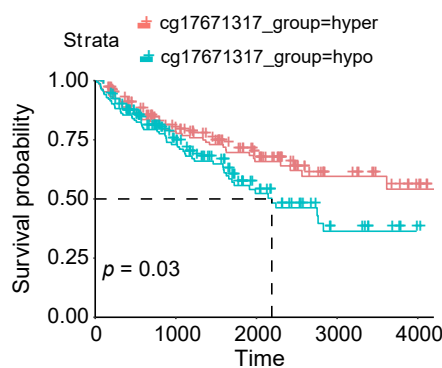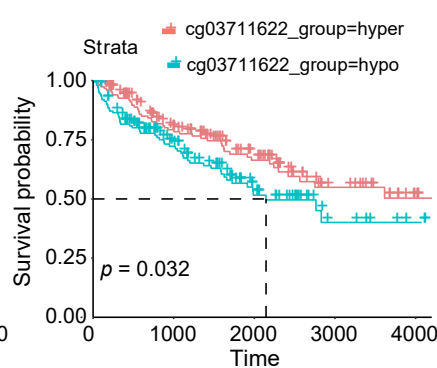**C**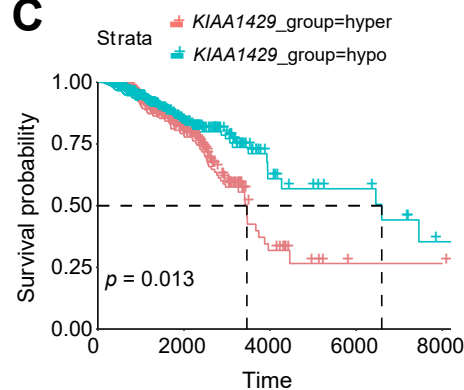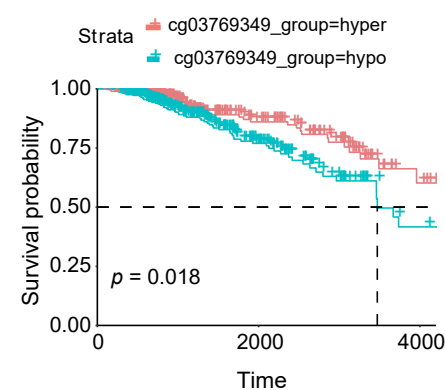

Supplement: Supplementary file 1 [file DataSheet_1.zip › Supplementary Figures/Figure S5.pdf]

**A**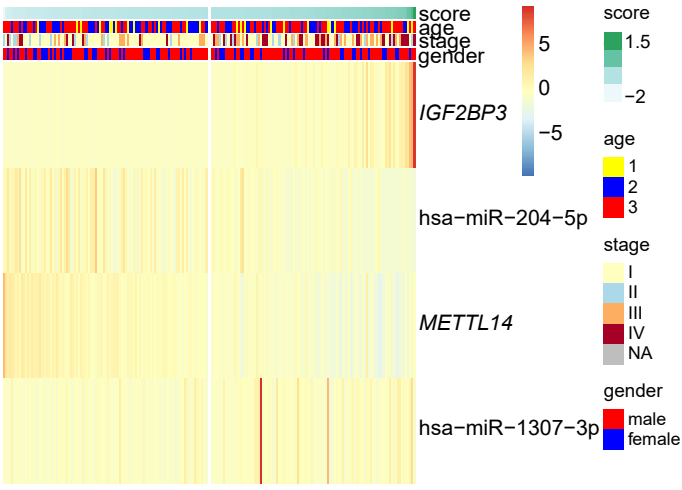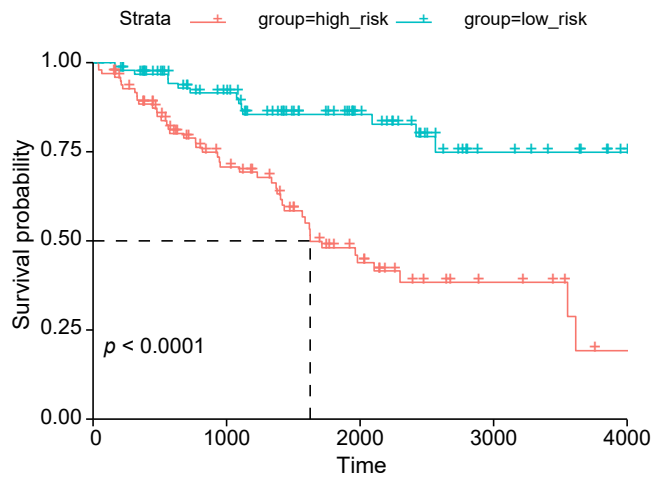**B**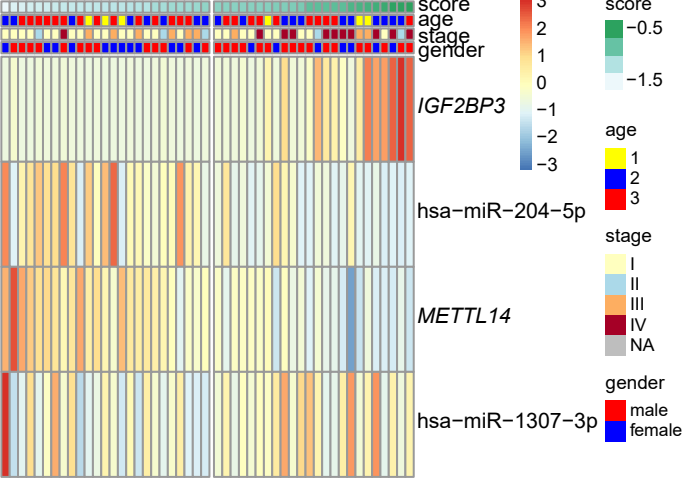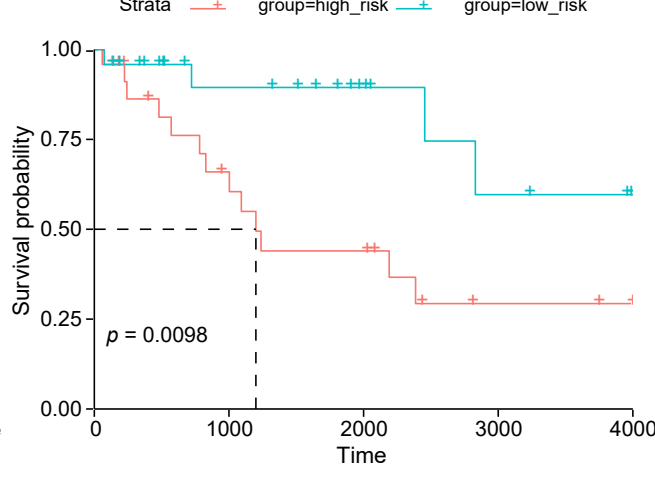

Supplement: Supplementary file 1 [file DataSheet_1.zip › Supplementary Figures/Figure S6.pdf]

**A**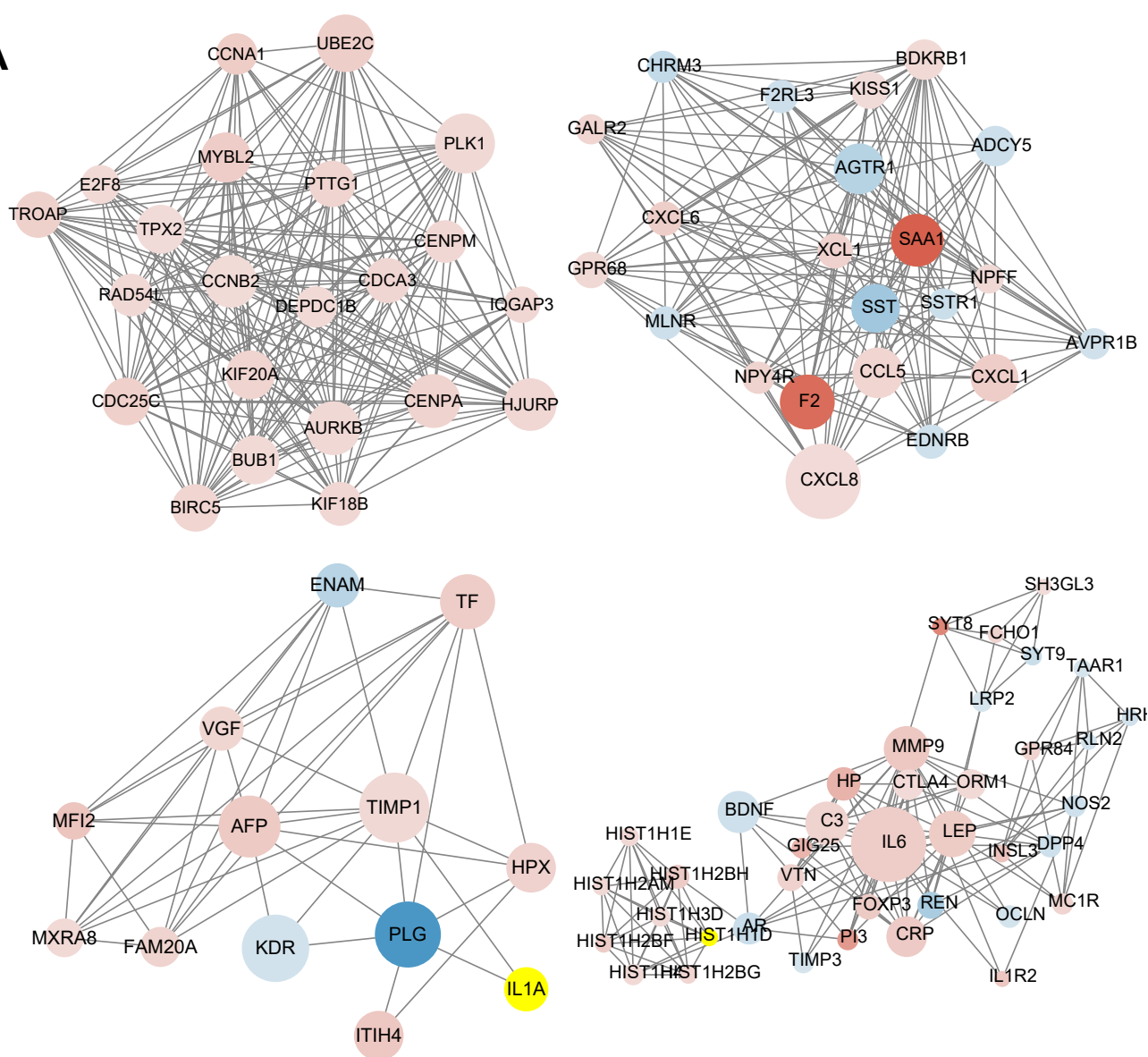**B**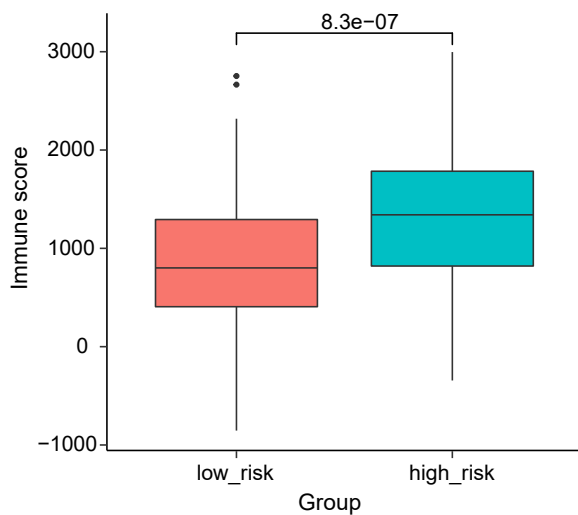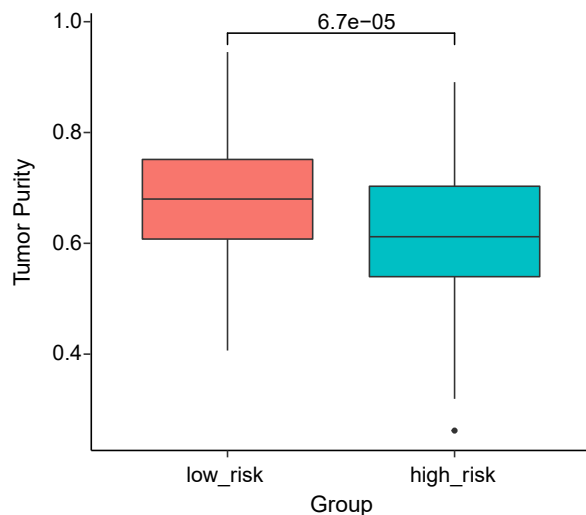

Supplement: Supplementary file 1 [file DataSheet_1.zip › Supplementary Figures/Figure S7.pdf]

**A**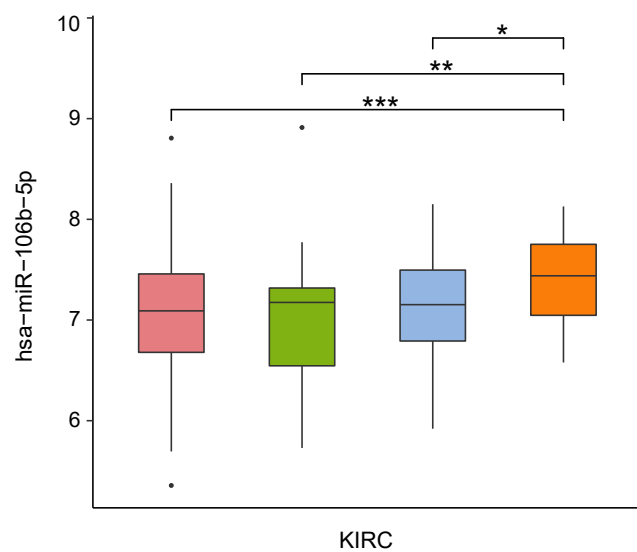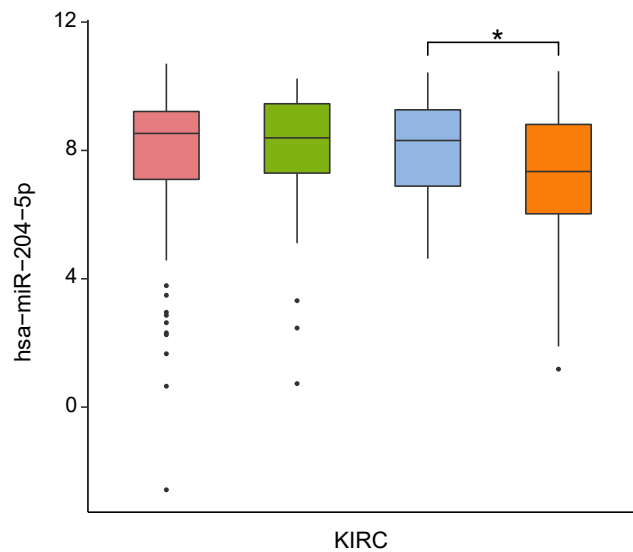**B**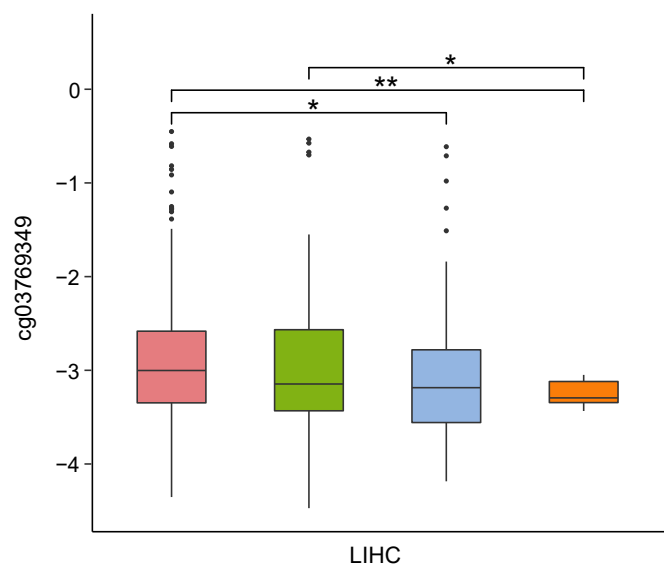

Supplement: Supplementary file 1 [file DataSheet_1.zip › Supplementary Figures/Figure S8.pdf]
